# Stress reactivity near birth affects nest building timing and offspring number and survival in the European rabbit (*Oryctolagus cuniculus*)

**DOI:** 10.1371/journal.pone.0246258

**Published:** 2021-01-29

**Authors:** Ildikó Benedek, Vilmos Altbӓcker, Tamás Molnár

**Affiliations:** 1 Department of Wild Biology and Ethology, Institute of Environmental Science and Nature Conservation, Szent István University, Kaposvár, Hungary; 2 Department of Nature Conservation and Environmental Management, Institute of Environmental Science and Nature Conservation, Szent István University, Kaposvár, Hungary; University of Tasmania, AUSTRALIA

## Abstract

The physiological response to stressors has great importance, and its variance has an adaptive role in the survival of individuals. This study describes the effects of stress-axis activation on maternal behavior during the birthing process (parturition) in captive rabbits (*Oryctolagus cuniculus*). In this species, chances of survival are strongly influenced by nest quality. Thus, maternal care is initiated with nest preparation in late pregnancy, which itself is subject to strict and complex hormonal regulation. Among these hormones, progesterone is one of the most dominant in the process of nest construction. We have demonstrated that its level is altered by the level of cortisol elevation in the animal in question, potentially having an influence on the preparation of the nest for the newborn kittens. We found that does that had a constant and un-elevated level of cortisol metabolite while delivering their litters performed better than those individuals that showed an increased corticoid response around parturition. The latter group exhibited a perceptible delay in the building of their nests, and in addition, further losses were also experienced in their already smaller litters. As the quality of the nest itself proved to be was in no way inferior to those of the other group, this higher kitten-mortality rate may be attributed to impaired maternal behavior. Individual variances in cortisol levels may also result in subtle changes in hormonal regulation, potentially affecting the expression of maternal behavior. We have concluded that the higher level of cortisol detected in more-sensitive does effectively disrupts the natural hormonal regulation involved in their nest-building processes.

## Introduction

Basic parental care, which enables offspring to survive during the period of parental dependence, is of vital importance in altricial species. Among mammals, maternal care prevails [1]. While numerous maternal care strategies exist, the strategy adopted is not simply a product of the number of offspring produced [2]. Multiple behavioral and physical elements constitute parental care and thus contribute to the development and success of the offspring [1,3]. Preparation of the nest cavity for offspring by the mother serves to exemplify this. Over 4500 known mammalian species construct burrows [4], and in a number of them, this process of burrow construction follows a specific and defined behavioral sequence [5,6].

The European wild rabbit is a nest builder, and lives under relatively intense pressure in its natural habitat from a predation standpoint, where it is the primary food source of more than 20 mammalian and avian predators [7,8]. Unlike many other herbivores [5,9] which spend a considerable amount of time together with their offspring, the maternal behavior of this particular species is limited to the time spent building the nest and one singular daily nursing visit. Any delay in nest construction may have an influence on the reproductive success of the mothers. Seltmann, et al. [10] reported that the rate of litter mortality around the time of birth for those mothers who began their in burrow-digging and nest-building late (usually subordinate does) increased despite having successfully completed all the steps for nest construction well within a 24 hour period.

The nest-building of the rabbit is composed of a time-controlled chain of behaviors which are regulated by both external (environmental) and internal (hormonal) factors. This complex process of nest building includes the digging of the burrow itself, the gathering of grass, and formulation of nest structure, such as lining it with the mother’s own fur [11–13]. External environmental factors, e.g., poor soil quality or lack of available grass, may limit both burrow-digging and nest-building. In the case of a sociable species such as the rabbit, the social environment may also have direct impact on the animal’s reproductive success [14,15]. In a number of cases, social stresses imposed by competition among females have negative consequences on reproduction [10]. These stress processes can be reflected though the measurement of glucocorticoid levels (GCM (glucocorticoid metabolites)), a well-established tool for measuring stress, and may also be evaluated non-invasively from feces through analysis of the degradation product of glucocorticoids [16].

Nest-building is a process, taking place under complex hormonal regulation, which determines the onset of a similarly complex series of behaviors it involves [11–13]; the major transitions in this process are attributed to progesterone, estrogen, and prolactin levels. The gestation period of the wild rabbit lasts for 31 to 32 days. Burrow-digging is triggered by a high β-estradiol and progesterone levels on the 25^th^-26^th^ day of gestation; the nest is subsequently prepared in this burrow, generally within 2–3 days, but sometimes only hours prior to parturition [10]. Along with the high level of β-estradiol, an eventual decrease in the level of progesterone effectively terminates the process of digging and simultaneously induces the gathering of nesting material and nest preparation. On the day prior to parturition, the level of prolactin rises nearly fivefold, causing fur shedding, thus enabling the mother to use it for lining the nest [17].

In the pregnant rabbit, serum levels of corticosterone (7.3 ng/ml) and cortisol (7.1 ng/ml) are identical until the last three days of pregnancy [18]. A decreasing progesterone level together with a parallel tenfold increase in cortisol between days 26–30 [18,19] has been described as an essential change in the third trimester of gestation, when the quantity of free glucocorticoid in the blood plasma rises due to higher-intensity glucocorticoid production [20] in the case of rabbits. Although still not yet confirmed in the case of rabbits, several other domesticated species (e.g., horses, sheep, cows, pigs, dogs) have been reported to exhibit elevated levels of maternal cortisol among other hormonal effects leading to parturition [21]. For gestating rabbits, the level of maternal cortisol reaches its maximum on day 30 (just before parturition) and immediately falls afterward [19]. It may be assumed that this high value is related to the initiation of parturition.

In addition to the ovaries and the placenta, the adrenal glands also play a significant role in the production of progesterone [22]. As early as 1971, Fajer, et al. [23] found that in female rats exposed to stress, the amounts of progesterone secreted in their adrenal glands and ovaries to be practically identical. The secretion of progesterone, similar to glucocorticoids, is controlled by the adrenocorticotropic hormone (ACTH) in the adrenal gland [22], making it more sensitive to stress. This may result in unique differences in the level of progesterone released by the animal.

The key factor in triggering nest-building behavior in females is the marked drop in progesterone levels towards the end of gestation [17]. Individual differences in cortisol levels may result in observable variation in the breeding process [24]. Notable individual variation was demonstrated in regard to the quality of the nest itself, subsequently affecting the successful outcome of reproduction, both in terms of size of the litter and the survival rate of the kittens [10]. It has also been documented in both humans [25] and domesticated pigs [26] that dramatic increases in glucocorticoid levels induced by maternal stress, lead to a decreased level of vitality in the offspring. In other rodents, adrenal progesterone release under stress is of notable significance. The present study aimed to investigate whether individual differences in cortisol release prior to parturition are a cause of significant differences in the level of progesterone during birth, and thus, the timing of the rabbit’s hay-carrying behavior. In our examination, we separated our subjects into two distinct groups based on their high (HR) and low cortisol responses (LR), occurring on the day of parturition. We hypothesized that a higher cortisol level would result in the prolongation of the progesterone levels, which would consequently cause a delay in the timing of the nest-building process, thereby affecting both litter size and mortality.

## Materials and methods

### Ethical approval

This research was approved by the Committee on the Ethics of Animal Experiments of the Szent István University Kaposvár Campus (permit number: MÁB/2-2/2019). The authors declare that all experiments were performed in accordance with approved guidelines and regulations.

### Experimental animals

Examinations were carried out on 30 females of mature European wild rabbits, 10 to 12 months of age, wherein their first parturitions were compared. One female did not produce offspring, and two gave birth to their kittens on the grid (out of the nest), and thus, their previously recorded data were excluded from further surveys. The rabbits taking part in the experiment were derived from grandparents who were caught in the wild, the offspring of which subsequently became familiar with being kept in cages and imprinted to humans after birth during the first week of lactation [27]. Due to this process, their inherent fear of humans was reduced, which was necessary to facilitate ease of handling [28].

### Experimental facility

Rabbits were mated naturally, and then placed individually into cages (floor area 60 x 60 cm, height 45cm) equipped with farrowing boxes (dimensions: 40 cm × 25 cm × 31 cm). The cages were made of welded wire mesh with hand-filled self-feeders and hayracks attached to the front. A rail-sliding tray made of galvanized steel provided a manure-receptical and was installed under each cage. The cages were placed in a room with a controlled temperature and photoperiod (15.4±1.6˚C, 16 hours of daily lighting), installed in two rows and separated with opaque plates. Individuals were able to hear and smell each other, but visual contact was not allowed. Drinking water came from hand-filled drip-waters. Commercial rabbit feed, water, and hay were made available to them ad libitum.

### Behavioral analysis

For the purpose of examining behavior in regard to grass collection, we provided dry grass over the standard amount of hay (approximately 100 g) to be used as nesting material 6 days before the expected time of parturition (from day 25).Behavior of the females examined was recorded with Sony HDR-XR cameras. Hay-carrying behavior can be detected quite easily as the doe collects the long pieces of grass in her mouth, which in protruding from both sides of the mouth, resemble a mustache. Similarly, the extraction of fur is also an easily-detectable behavior, observed when the female begins pulling out fur from its chest and thighs. Inception times for the collection of grass and fur extraction were examined by instantaneous sampling. At hourly intervals, we observed and recorded whether the females had begun carrying hay in their mouths or whether they had started to pull on their fur.

Approaching the day of parturition, the farrowing boxes were checked every 2–3 hours. Once new-born kittens were discovered, a sliding door separating the farrowing chamber from the cage was accessed to allow removal of the kittens. Next, kittens were handled through the top opening, the total number of kittens was recorded and among them, the number of live kittens.

Nests were categorised according to the following method: once the kittens reached 21 days of age, the nest was removed from the farrowing boxes. As the bottom of the nests had become wet from urine it was necessary to dry them out first. Once dry, the total mass of the nests was weighed, and the fur and blades of grass were arranged evenly in order to get a homogeneous mix. Ten samples were taken out of this amount (a pinch weighed appr.1-1.5 g). Every sample was separated into hay and fur and weighed individually on Sartorius scales in grams to two decimal points. This process produced the hay vs. fur ratio in the ten samples, enabling us to estimate the total hay and fur mass for the entirety of the nest (S1 Table).

### Hormone assessment

Although the primary stress hormone in the European rabbit is corticosterone, not cortisol, the cortisol levels were measured in the present study since it significantly increases in the last three days of the pregnancy [19]. Progesterone and cortisol levels were measured from feces based on their breakdown products (GCM) [29,30]. Fecal samples were collected every 24 hours (at 20:00 EET) beginning from day 28 of gestation until the day following parturition. Contamination of the feces with urine was prevented with a mesh placed in the tray under the cage. This allowed the feces to remain on the mesh while the urine flowed into the tray. Once a sample was collected, the trays underneath the cages were replaced with new, completely clean trays. Finally, fecal data was collected on that day, 2 days prior, and the day following parturition, were included in the analysis. Samples were stored at -20°C until extraction.

In addition to the collected samples, additional samples were taken to validate the cortisol measurement method. Ten mothers were exposed to social stress by placing two individuals in a transport box (30 * 30 * 40 cm) for one hour. The animals were removed from the box and immobilized by hand to collect blood samples. Subsequently, 2 ml of blood was taken from the ear vein (needle size 21G) within 2 minutes. The samples were centrifuged at 3,000g for10 min, and plasma was separated and stored at -20°C until further measurement. Twenty-four hours after blood collection, a fecal sample was taken as described above and stored at -20°C.

The protocol for fecal extraction was adapted from previously published methods [29]. After freeze-drying, the samples were ground, homogenized, and mixed thoroughly. 200 mg of dry-feces was then placed in a glass vial and 1.6 ml 80% methanol and 200 μl distilled water were added to extract the hormone metabolites. The vials were capped and vortexed for 30 minutes. Samples were then centrifuged (2450 rpm, 20 min, 4°C) and the supernatant was poured off and stored at– 55°C. At the time of use, samples were dried in a chamber (Binder) and reconstituted with ASB buffer at a 1:1 dilution rate. Unlike the original method [29] where a an EIA with a highly specific antibody to a fecal corticosterone metabolites was used, a RIA method was used for cortisol and progesterone, which were developed for hormone determination in the plasma of food animals using tritium labeled hormones (cortisol and progesterone-1,2,6,7-3H(N)) as well as highly-specific polyclonal antibodies raised against cortisol-21-HS-BSA and 11αOH progesterone11HS:BSA in rabbits. The validation of the RIA method for the fecal metabolites was performed by determining the relationship between the measured levels in the serum and the feces samples. The binding parameters of the cortisol antibody were the following: specific activity >4 TBq/mmol, the lower limit of detection: 15 fmol, affinity: K_A_ = 3.0x10^10^ l/mol. The reconstituted antiserum binds 45% of 10 000 cpm H^3^ labelled cortisol. Cross-reactivity of the antiserum can be seen in S2 Table. Intra-assay variation was determined to be < 5 CV%, for both cortisol and the progesterone. Inter-assay variation was 9.63 CV% for cortisol, and 1.68 CV% for progesterone. Sensitivity was 0.433 ng/mg for cortisol, and 0.199 ng/mg for progesterone. For the purpose of determining comparability between cortisol standards in rabbits and the concentration of cortisol in their feces, a high-concentration fecal sample was diluted in a sequential manner. The relationship between fecal cortisol and the standard concentration curve was determined through linear regression, the correlation coefficient was 0.998 (r) and the model for it was based on the y = 0.946x + 4.781 equation.

### Statistical analysis

For examining differences in cortisol levels during gestation and at the time of parturition and their correlations with progesterone levels at time of parturition, and the correlation between the plasma cortisol and FCM levels, linear regression was applied. Arrangement into groups based on differences in cortisol levels during gestation and at the time of parturition was carried out using cluster analysis (k-means cluster). The number of clusters was two, and the number of iterations was limited to 10. The differences in progesterone between the two groups were examined using a two-sample Student’s t-test both during gestation and at time of parturition, and at the time of hay stacking, for the purpose of determining the quality of the hay and fur collected and the number of the kittens born. The time of fur-stacking did not show a normal distribution; as such a non-parametric Kruskal-Wallis test was used. Kitten mortalities by group were compared with a Chi^2^ test. Normality of the data collected was checked using the Shapiro-Wilk- test, while their homogeneity was confirmed with the Levene- test. Differences among the groups were accepted to be statistically significant at a level of P<0.05. Statistical analysis was carried out with the SPSS 11.5 software.

## Results

The plasma cortisol levels and the fecal cortisol metabolite levels showed a significant correlation (R = 0.8). The regression model describing the relationship between the two parameters is y = 530.593x-1.221 (P = 0.005, R2 = 0.64, S1 Fig).

**Fig 1:**
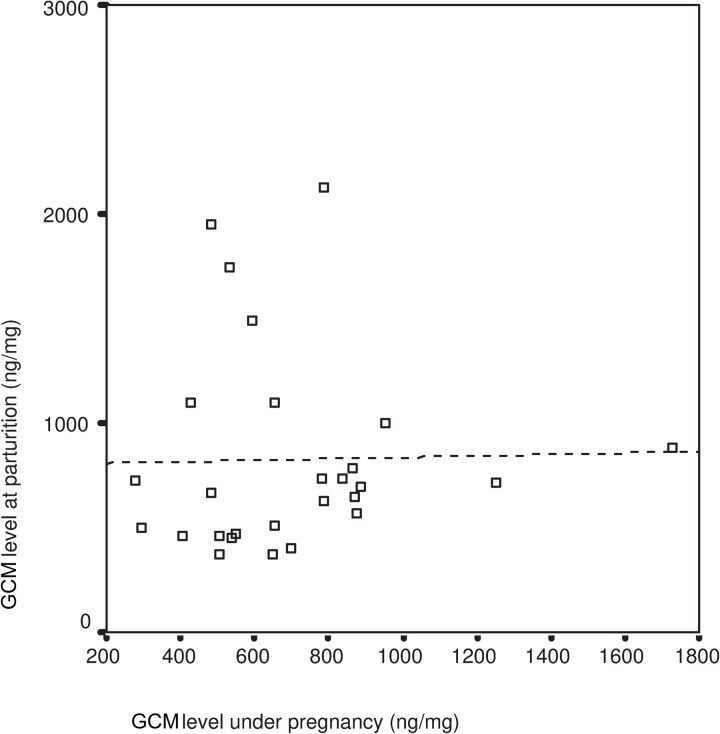
Relationship between GCM during pregnancy and on day of parturition GCM. GCM-Glucocorticoid Metabolites, linear regression: R = 0.022, P = 0.914.

**Fig 2:**
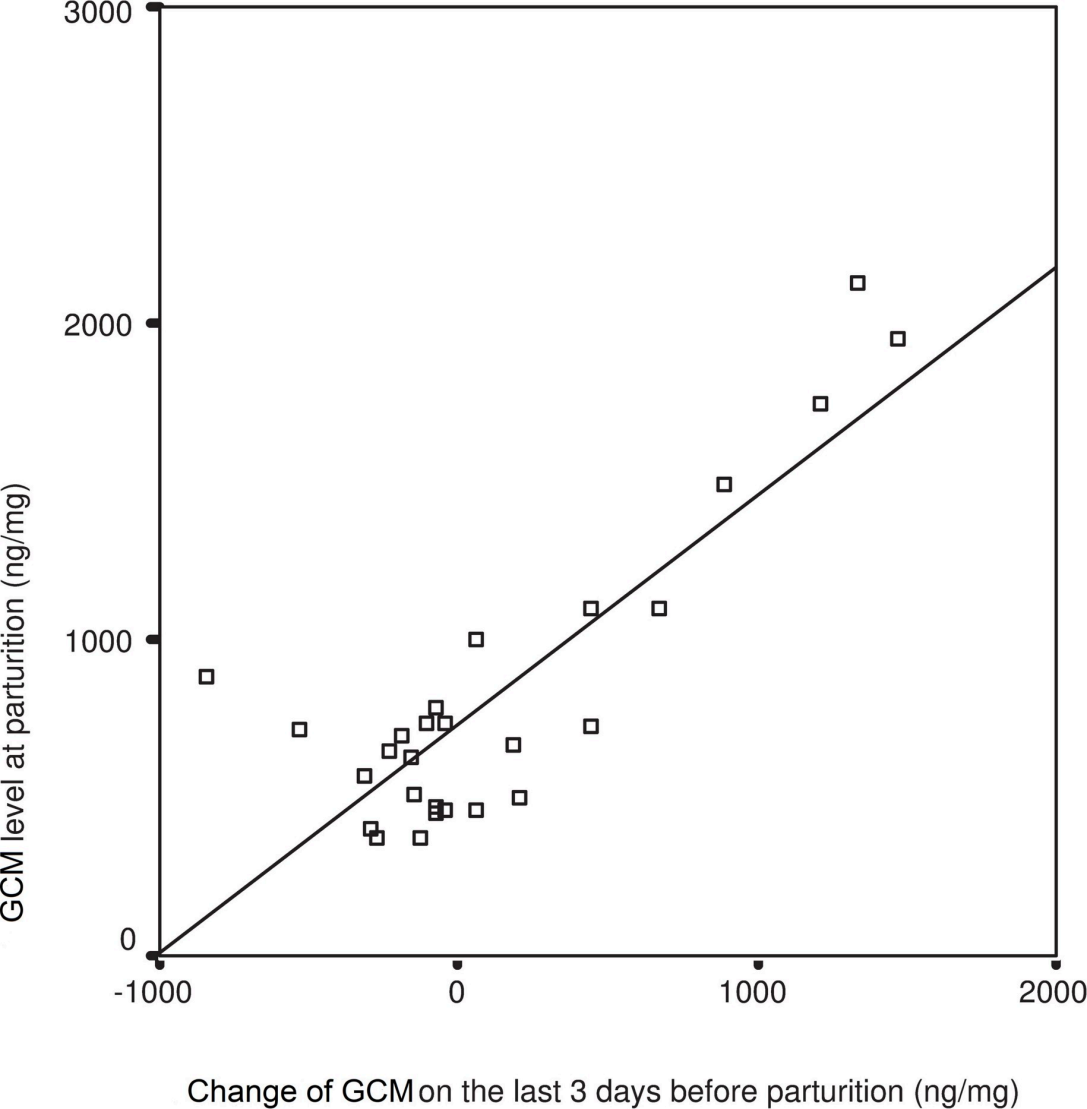
Relationship between GCM on day of parturition and the changes in GCM during last 3 days of pregnancy. GCM-Glucocorticoid Metabolites, linear regression: R = 0.843, F = 61.617, P = 0.001.

The cortisol level measured at the time of parturition showed no correlation with the cortisol levels measured during gestation (R = 0.022 F = 0.012 df = 26 p = 0.914) (Fig 1). However, a significant correlation between the rise in cortisol levels in the last three days of gestation and the cortisol levels measured on the day of parturition was observed (R = 0.843 F = 61.617 df = 26 P = 0.001) (Fig 2).

**Fig 3:**
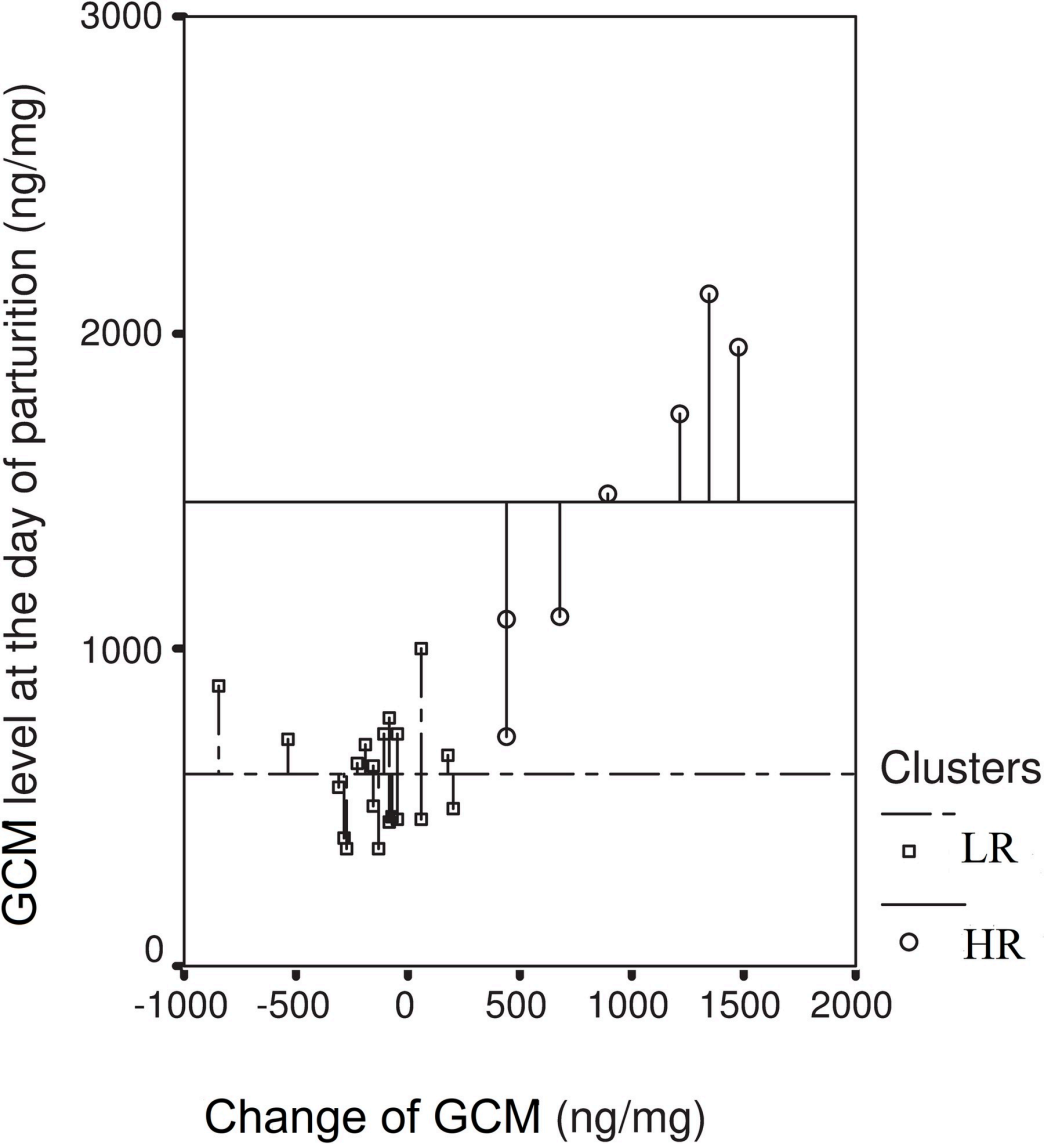
GCM levels on the day of parturition within two groups distinguished by changes in their GCM levels during the 3 last days before parturition. LR- Low-response group: 20 individuals showing either a decreasing or slightly-increasing value, HR-High-response group: 7 individuals showing a substantial increase.

The average cortisol level at the time of parturition was 826.01±479.29 ng/mg (Fig 3) in the animals examined. The cluster analysis which was performed on the basis the difference between cortisol levels measured three days before parturition and on the day of parturition itself, divided the animals into two groupings. In some individuals (HR group), a significant increase in cortisol levels took place (925.48±424.75 ng/mg); applying to 7 rabbits (average cortisol level at parturition: 1462.09±511.37ng/mg). In the LR group, consisting of 20 rabbits (average cortisol level at parturition: 603.38±174.74 ng/mg), cortisol levels demonstrated a decrease (-152.09±236.55 ng/mg).

**Fig 4:**
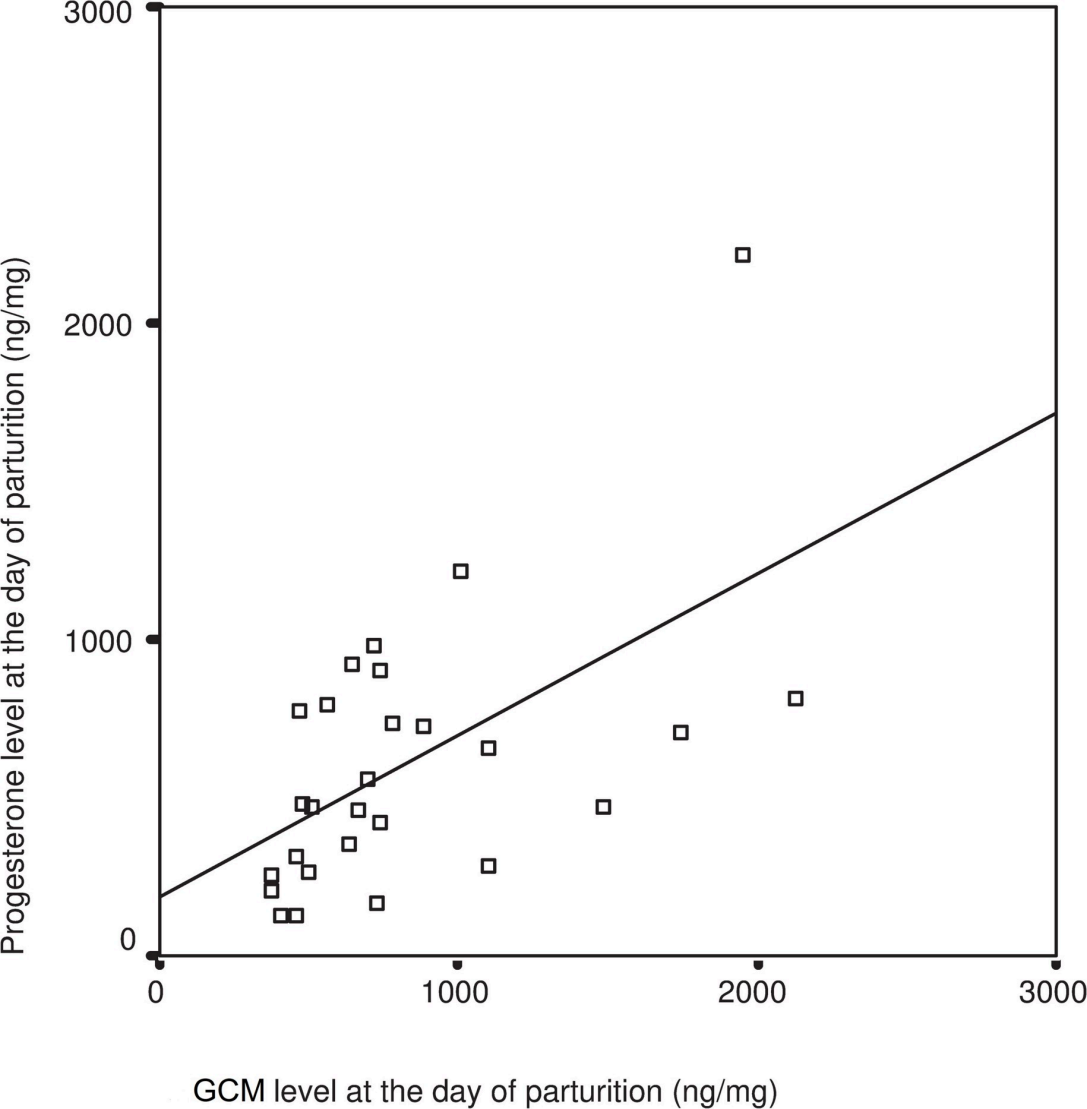
Relationship between GCM and progesterone values on day of parturition. GCM-Glucocorticoid Metabolites, linear regression: R = 0.567 F = 11.871 P = 0.002.

Cortisol and progesterone measured on the day of parturition showed a moderate correlation across all does(R = 0.567 F = 11.871 P = 0.002) (Fig 4).

**Fig 5:**
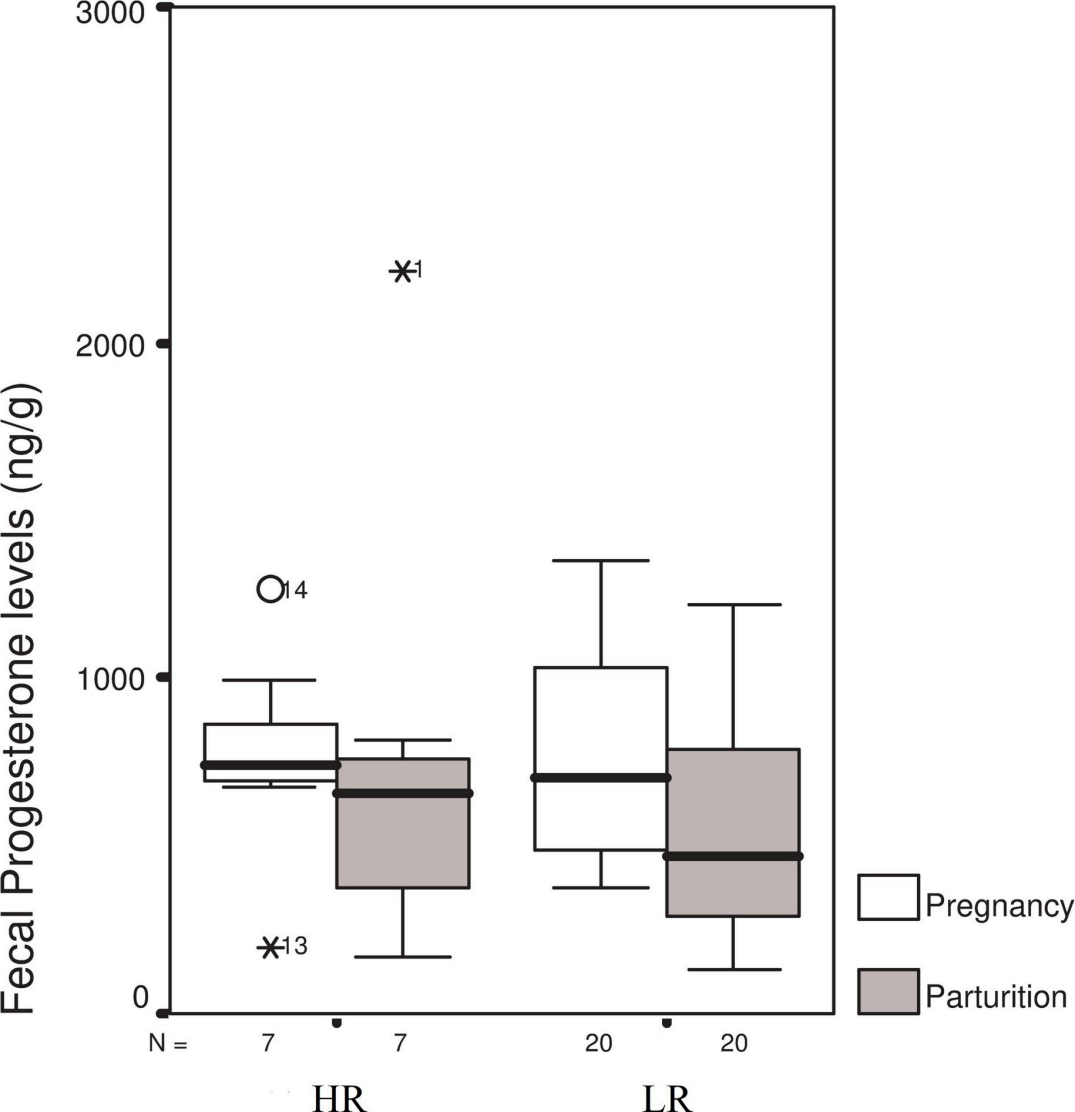
Changes in progesterone levels measured during pregnancy and time of parturition in the treatment groups. LR- Low-response group, HR-High-response group, two-sample t-test, t = 1.086 df = 25 P = 0.288.

The level of progesterone during gestation did not differ between the two groups (HR and LR). The average progesterone level showed a 28% decrease in the LR group. However, this change was lower in the HR group, resulting in 554.77±310.68 and 759.89±682.25ng/mg progesterone levels on the day of parturition in the LR and HR groups, respectively. Differences between the groups were not significant (t = 1.086 df = 25 P = 0.288) (Fig 5). A significant difference was found, however, in the starting time for hay collection(t = -2.238 df = 25 P = 0.034), where the mean scores of both cortisol elevation-based groups averaged at 26.14 ± 31.39 hours (HR) and 76.10 ± 55.57 hours (LR). No significant timing difference was indicated in regard to fur collection (Kruskal-Wallis test Chi^2^ = 1.642 P = 0.200), although the HR group tended to start collecting hay earlier (3.42±3.82 hours vs. 20.95±32.86 hours for the LR group).

**Fig 6:**
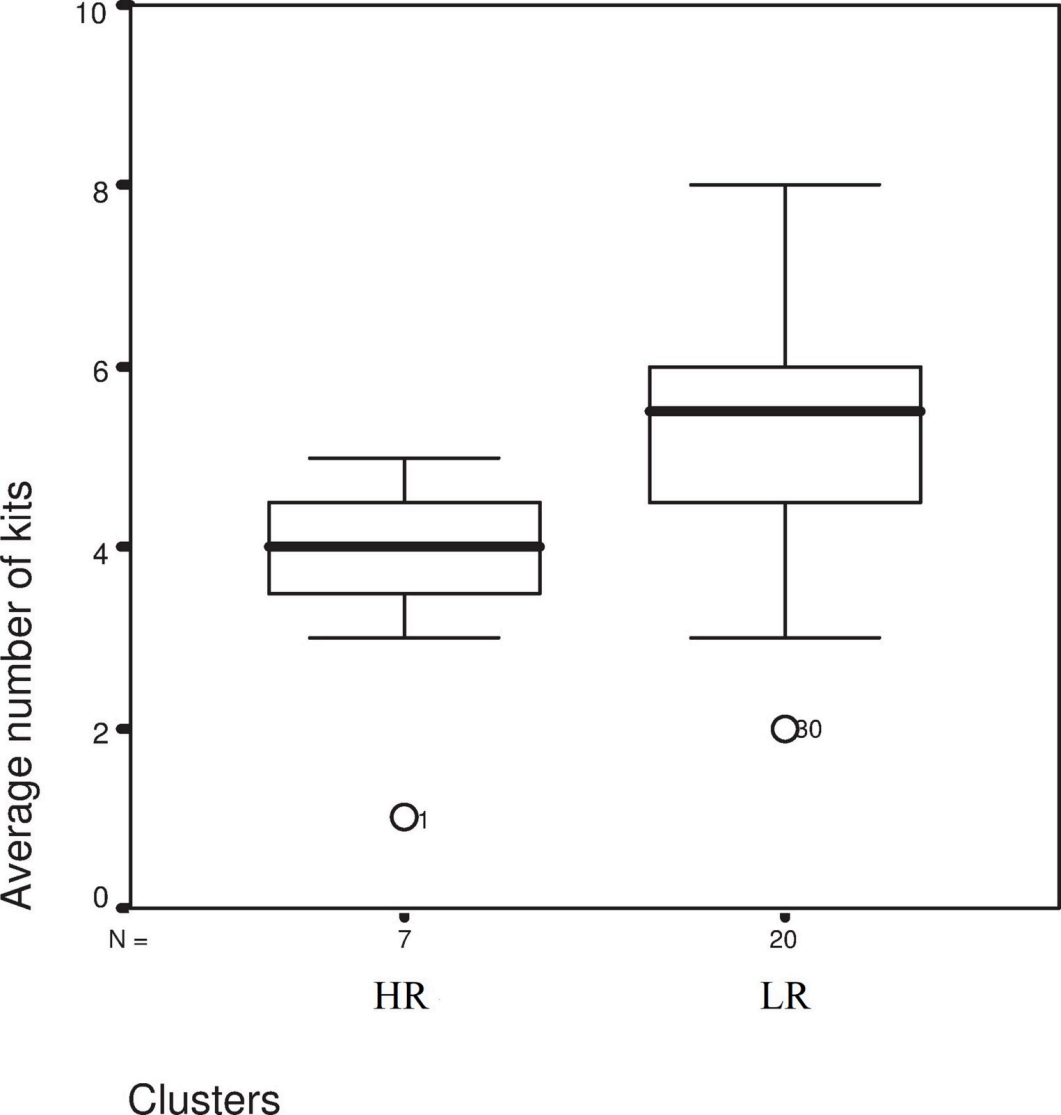
Number of kittens in the two treatment groups. LR- Low-response group, HR-High-response group, Chi^2^ test, Chi^2^ = 5.092 df = 1 P = 0.024.

Amounts of hay collected did not differ significantly (t = -0.558 df = 25 p = 0.582) between the two groups (HR: 146.21±37.89g vs. LR 160.10±61.51g). The amount of built fur (HR: 10.91±6.15g vs. LR 14.20±13.18g) was very similar in each group (t = -0.631 df = 25 P = 0.534). A significant difference was found in cortisol elevation-based groups regarding the average number of kittens born (t = -2.185 df = 25 p = 0.038), as the number of kittens born (Fig 6) was lower in the HR group. Additionally, perinatal mortality (birthday) was significantly higher (Chi2 = 5.092 df = 1 P = 0.024) in the HR group than in the LR group.

## Discussion

We studied how behavioral patterns and hormonal changes in captive rabbits interact during the breeding period in order to gain greater understanding into why significant variation exists in their breeding success. We found that although certain social interactions were prevented through individual caging, variation occurred in both their behavior as well as in those underlying hormonal changes characteristic to certain phases of the breeding process.

We found that changes in the level of cortisol in the last three days of gestation was a good indicator of the sensitivity of the individual subject. However, mid-gestation cortisol levels (see Figs 1 and 2) did not allow us to predict the subject’s level of cortisol elevation at the time of parturition. This result matches findings of Edwards and Boonstra [20] revealing that glucocorticoid levels stay low until the end of gestation. Cabezas, et al. [31] has also demonstrated that European rabbits display large individual variation in their glucocorticoid levels, as measured in their serum or their GCM’s. This noteworthy difference between the rabbits indicates that individuals may react to stressful situations quite differently.

Even small differences in hormonal changes which are responsible for triggering internal chain processes around the birthing process can result in differences in reproductive performance. The change in cortisol levels (Figs 4 and 5) observed in our rabbits corresponded to the progesterone production of the pregnant animal, as levels of cortisol and progesterone change on the day of parturition. We may assume that the positive correlation between progesterone and cortisol around the time of parturition was due to the higher cortisol elevation induced secretion of progesterone stored in the adrenal gland. This is due to the fact that not only ovaries, but also the adrenal glands, yield a significant amount of progesterone in lagomorphs, as well as in humans. Fajer, et al. [23] described, as early as 1971, that ACTH regulates the production of progesterone in the adrenal gland. In female rats exposed to mild stress, progesterone secretion becomes as intensive in the adrenal gland as in the ovary. A similar result was published by Yoshida and Nakao [32] on milk cows and by Beaulieu-McCoy et al. [33] on the Californian sea lion (*Zalophus californianus*), where a positive correlation was found between cortisol and progesterone levels subsequent to a stress event. This may be explained by considering the metabolic pathway in question, as progesterone is a precursor of cortisol. Acute stress-related progesterone secretion can be explained through the limited activity of an enzyme (cytochrome P450-C21 enzyme), which is partially responsible for building cortisol from progesterone. As part of the progesterone is released into the bloodstream before turning into cortisol, the concentration of circulating progesterone also increases [33].

We divided our does into two groups based on their cortisol-level change measured in the last three days of gestation. Low-response rabbits (LR group), showing no increase in GCM levels at the time of parturition, and exhibited normal maternal behavior, from collection of nesting material to the creation of the nest. High-response rabbits (HR group), characterized by an increase in their GCM levels around the time of parturition, were delayed by approximately 50 hours in regard to the hoarding of nesting material. This result supports the conclusions of a Seltmann, et al. study [10], wherein stress caused by social instability and frequent competition between groups appeared to bring about delayed burrow-digging and nest-building behavior in the subordinate females, supposedly as a result of having elevated glucocorticoid levels. In our case, the rabbits were caged individually, and nesting materials were freely available to them, ad libitum, resulting in the minimalisation of social stress. This allowed us to observe with clarity that certain females reacted to birthing (a new and, unexpected situation) more sensitively, and consequently, responded to their higher cortisol elevation with a delayed behavioral pattern. Gonzalez-Mariscal, et al. [11] described that the beginning of hay-collecting and nest-building is signaled by a drop in the level of progesterone. A higher elevation in cortisol levels, however, results in a progesterone level which also stays high, and this may delay the collection of hay. Hoffmann, et al. [34] also suggested that a change in the secretion of progesterone might cause a time shift in the burrow-digging of European rabbits kept under laboratory conditions. They found a positive correlation between burrow-digging and elevated progesterone levels as well as increased intraspecific aggressive behavior in response to administered progesterone. A similar phenomenon was described by Farkas, et al. [35], who successfully identified two peaks in relation to the gathering of nest material in domesticated rabbits. The first peak designating the carrying of nesting material, was on days 27 and 28, nearly identical to the normal (LR) does in our experiment. The other peak of nest building culminated on the day of parturition, and corresponds to the results of our sensitive (HR) group. Our results highlight the fact that it is the individual sensitivity of the animal which decisively affects the behavioral response. More sensitive individuals react to emerging challenges with an increased glucocorticoid level, intense cortisol production also leading to a rise in progesterone levels.

Delays in nest-building were observable in individuals showing elevated GCM levels in response to parturition. Nonetheless, these individuals were still capable of building nests which were identical in quality and structure to the nests of does with less-elevated cortisol levels, who had begun their nest-building significantly earlier. Despite the short time frame involved (the final day), as well as our previous assumptions, neither the quantity of hay collected nor the fur extracted for the purpose of building the nest differed between the two cortisol-response groups. In domesticated rabbits kept under laboratory conditions, Denenberg, et al. [36] found that nests made earlier were of better overall quality, when the basis of scoring was limited to the timing of hay–carrying and, the presence of grass and fur provided. In this system, nests built on the last day received low-quality scores. In our study, we demonstrated nest composition to be the same in this regard. Unlike in our experiment, under natural circumstances in the wild, nest materials are not always at the animals’ disposal.

We also found that does with higher GCM values around the time of parturition delivered smaller litters, and there were also further losses within these litters. In the European rabbit, 54% of pre-weaning mortality occurs within the first 12 hours, and 70% within the first week of the kittens’ life [37]. There was a significant difference between our groups (differentiated on changes in cortisol levels), as litter size was by 1.44 kittens larger in the LR group. Additionally, with a higher cortisol level around the time of birth, offspring mortality was 9.6% higher in the HR group than in the LR litters. Rödel, et al. [38] described litter size, birth body mass, and temperature as the most significant factors influencing kitten mortality. However, it was also found that nest mortality was higher in subordinate females exposed to social stress. This causal explanation is congruent with our findings as well, with the exception that our animals were individually caged and therefore less exposed to social stress. Furthermore, Seltmann, et al. [10] attributed the increased kitten mortality observed in cases of delayed burrow-digging and nest-building to be the result of stress induced by intra-sexual competition. On the basis of our present study, we offer a different causal explanation. In the ground squirrel (*Urocitellus richardsonii*), higher total cortisol during gestation resulted in a lower litter size [39]. It also affects the sex ratio of the offspring through a positive relationship between maternal testosterone and GCM, based on the stimulating effect of ACTH on the adrenal gland which is the primary source of testosterone in females [40]. In our study, the analogous stimulatory effect of ACTH influences the timing of nest formation through progesterone release. Specifically, in addition to the exclusion of social stress, individual variance in cortisol levels point to subtle changes in hormonal regulation and may therefore affect both the timing as well as the successful outcome of maternal behavior.

## Supporting information

S1 Fig Relationship between plasma cortisol and fecal cortisol metabolites (FCM) collected from ten rabbit does. (TIF)

S1 Table The raw data of the experiment. (XLS)

S2 Table Cross reactivity of the antiserum used for GCM measurement. (PDF)
