# Supplementary figures and images for: Stress reactivity near birth affects nest building timing and offspring number and survival in the European rabbit (Oryctolagus cuniculus)
Source: PLoS One. 2021 Jan 29;16(1):e0246258. doi: 10.1371/journal.pone.0246258 (PMC7845978; doi:10.1371/journal.pone.0246258)

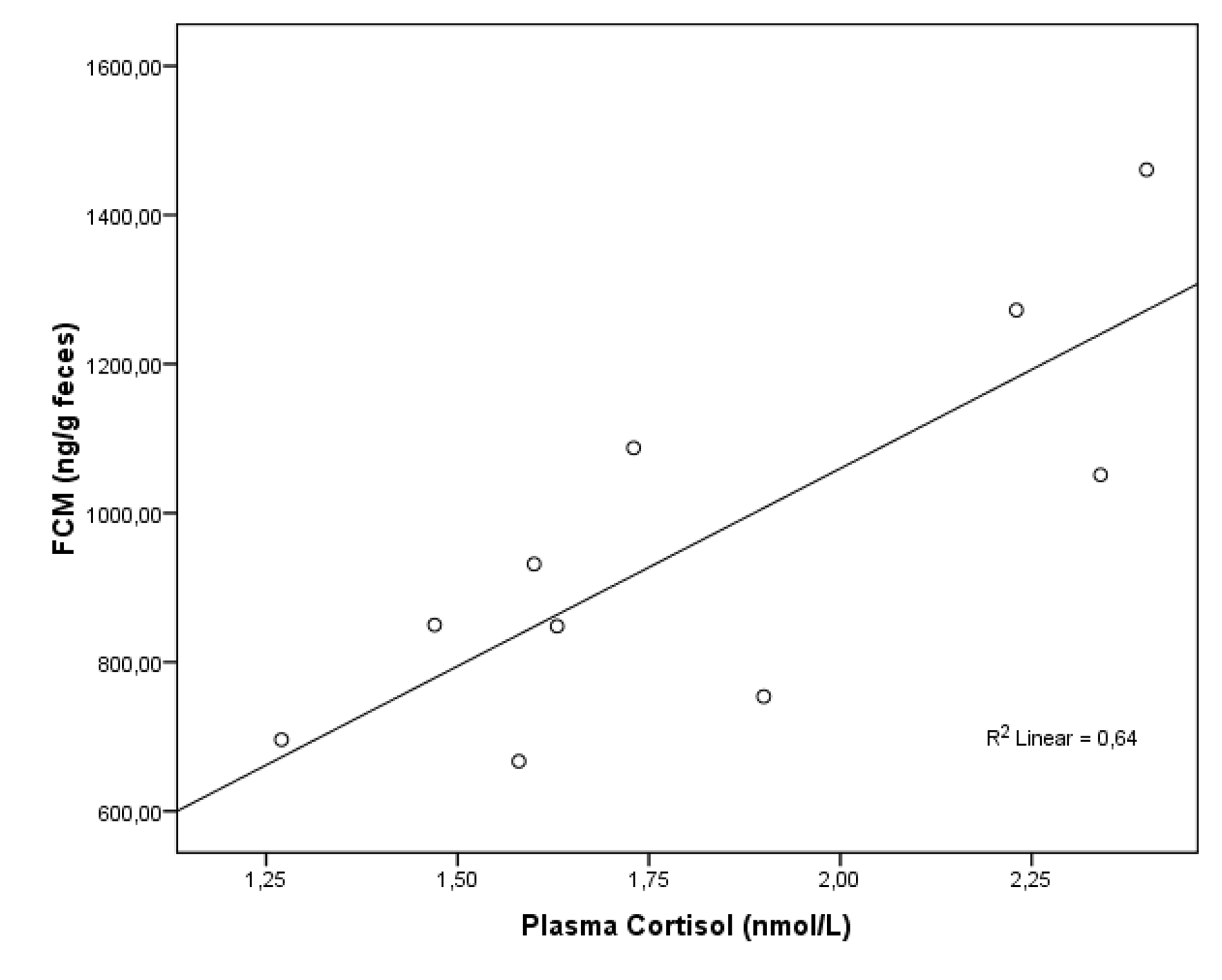

Supplement: S1 Fig — (TIF) [file pone.0246258.s001.tif]
