# Supplementary material for: Stress reactivity near birth affects nest building timing and offspring number and survival in the European rabbit (Oryctolagus cuniculus)
Source: PLoS One. 2021 Jan 29;16(1):e0246258. doi: 10.1371/journal.pone.0246258 (PMC7845978; doi:10.1371/journal.pone.0246258)
Supplement: S2 Table — (PDF) [file pone.0246258.s003.pdf]

Table S2

Cross reactivity: (B/B_0=.5)	%
Cortisol (Hydrocortisone)	100
Deoxycortisol	6.4
Corticosterone	19
Deoxycorticosterone	.45
Prednisolone	9.5
Cortisone	
Cholesterol	<.0001
Pregnenolone	<.0001
17αOHpregnenolone	<.0001
Progesterone	2.6
20αdihydroprogesterone	.54
17αOHprogesterone	5.7
11αOHprogesterone	.066
Dehydroepiandrosterone (DHA)	<.0001
5αdihydrotestosterone	.015
5ßdihydrotestosterone	.024
Androsterone	<.0001
Androstenedione	.191
Testosterone	.38
17αmethyltestosterone	.31
Ethisterone	<.0001
Estradiol17ß	<.0001
Estradiol17ß3benzoate	<.0001
17αethynilestradiol17ß	<.0001
17αethynilestradiol3methyl ether	<.0001
Estradiol3methyl ether	<.0001
Estradiol17α	<.0001
Estrone	<.0001
Estriol	<.0001
